# Quantifying human mobility behaviour changes during the COVID-19 outbreak in the United States

**DOI:** 10.1038/s41598-020-77751-2

**Published:** 2020-11-26

**Authors:** Yixuan Pan, Aref Darzi, Aliakbar Kabiri, Guangchen Zhao, Weiyu Luo, Chenfeng Xiong, Lei Zhang

**Affiliations:** Department of Civil and Environmental Engineering, University of Maryland, 1173 Glenn Martin Hall, College Park, MD 20742 USA

**Keywords:** Epidemiology, Health policy, Lifestyle modification, Viral infection

## Abstract

Since the first case of the novel coronavirus disease (COVID-19) was confirmed in Wuhan, China, social distancing has been promoted worldwide, including in the United States, as a major community mitigation strategy. However, our understanding remains limited in how people would react to such control measures, as well as how people would resume their normal behaviours when those orders were relaxed. We utilize an integrated dataset of real-time mobile device location data involving 100 million devices in the contiguous United States (plus Alaska and Hawaii) from February 2, 2020 to May 30, 2020. Built upon the common human mobility metrics, we construct a Social Distancing Index (SDI) to evaluate people’s mobility pattern changes along with the spread of COVID-19 at different geographic levels. We find that both government orders and local outbreak severity significantly contribute to the strength of social distancing. As people tend to practice less social distancing immediately after they observe a sign of local mitigation, we identify several states and counties with higher risks of continuous community transmission and a second outbreak. Our proposed index could help policymakers and researchers monitor people’s real-time mobility behaviours, understand the influence of government orders, and evaluate the risk of local outbreaks.

## Introduction

Since the World Health Organization (WHO) declared COVID-19 a pandemic, the novel coronavirus and its impact on countries and regions worldwide has drawn much research attention^1–3^. As one of the major non-pharmaceutical interventions, social distancing—or physical distancing—is considered an effective way to reduce COVID-19 infections. In the United States, government agencies have taken actions to promote social distancing and mitigate the spread of COVID-19, such as educating the public on the importance of social distancing, closing non-essential businesses, and issuing mandatory stay-at-home orders. Beginning in late April, phase-by-phase reopening plans have been gradually deployed in different regions to help people resume their normal life. However, the pandemic has prompted many questions. How do people react to government actions and perform social distancing? What is the reopening readiness of each region? How can we measure the risk of a second outbreak? This paper proposes a Social Distancing Index (SDI) based on mobile device location data to reveal people's mobility patterns in response to the COVID-19 outbreak, social distancing policies, and reopening plans. A high score of SDI indicates that people are practicing more social distancing while a low score indicates less social distancing. The objective of this study is to provide more insight into people’s movements, which could help policymaking for public health and accommodate epidemic modelling improvements.

People’s actual behaviours in response to interventions are of great importance in modelling transmission dynamics. Existing studies on impact assessment of control measures mainly estimate related modelling parameters by Markov Chain Monte Carlo (MCMC)^4^; utilize the simulation models to estimate contact network based on a synthetic population^5^; estimate the contact patterns using survey data, modelling and simulation^6,7^; and collect people’s behaviour reactions through dedicated surveys^8^. We found that there is a lack of timely contributions from real-world observations. Meanwhile, studies that evaluate the mobility changes during the pandemic from real-time and real-world observations mainly focus on a single indicator: distance travelled. The topics include the development of a social distancing scoreboard at the national, state, and county level^9^, the direct impact of stay-at-home mandates^10^, and mobility patterns by income distribution^11^, etc^12–14^. A single metric, such as distance travelled, is not sufficient to capture the mobility changes and to portray individual efforts in social distancing. Considering the various measurements of human mobility patterns, such as number of trips made per person and an origin and destination matrix that displays the trips made between regions, an inclusive index is needed to simplify the information regarding different dimensions of human movement. An index also makes it easier for stakeholders to communicate with each other^15^, especially when navigating the challenges of the COVID-19 pandemic.

To properly design the structure of the Social Distancing Index (SDI), we have reviewed the existing indices from various fields. Based on our findings, there are two main types of indices: category-based indices and score-based ones. The category-based indices explain the proposed objective by categories. For example, the Pandemic Severity Index (PSI) classifies the case fatality ratio (CFR) of a disease into five categories (from one to five)^16^, and the Modified Mercalli Intensity Scale evaluates the severity of an earthquake by categorizing it into twelve levels from I to XII^17^. On the other hand, score-based indices usually define a score from zero to one hundred to differentiate objectives and rank them in order. For example, U.S. News State Ranking creates a score that covers eight topics on people’s needs in each state and assigns different weights to those topics based on the survey data^18^. Bloomberg Global Health Index is another score-based index that ranks countries in terms of healthiness by giving them a rate between zero and one hundred^19^. In short, category-based indices are usually built upon a single variable and the score-based ones are more capable of integrating multiple metrics to be more informative.

In this study, we incorporate five basic mobility metrics in the score-based SDI to comprehensively evaluate people’s behaviours in social distancing, e.g., number of personal trips (work and non-work) made daily and percentage of out-of-county trips. These metrics are generated from mobile device location data by data fusion and analytics. Mobile device location data is an emerging data source that provides insight into real-time human mobility patterns through a large sample size and continuous observations. Researchers have utilized such data to understand individual human mobility patterns^20^, to understand the spreading patterns of mobile phone viruses^21^, to explore social ties and link prediction^22^, and to evaluate the impact of human mobility on epidemics^23–25^. In response to the COVID-19 pandemic, researchers have also discussed how mobile device data could help them understand the spatiotemporal distribution of the disease^26^, and help policymakers control infection, optimize policymaking, and evaluate the effectiveness of released policies^27,28^ without overlooking the privacy issues surrounding digital data^29^. Here, we introduce mobile device location data as an appropriate and functional data source to measure the profound impact of COVID-19 and facilitate policymaking based on real-world and real-time observations.

## Results

### Effectiveness of Social Distancing Index (SDI)

**Figure 1:**
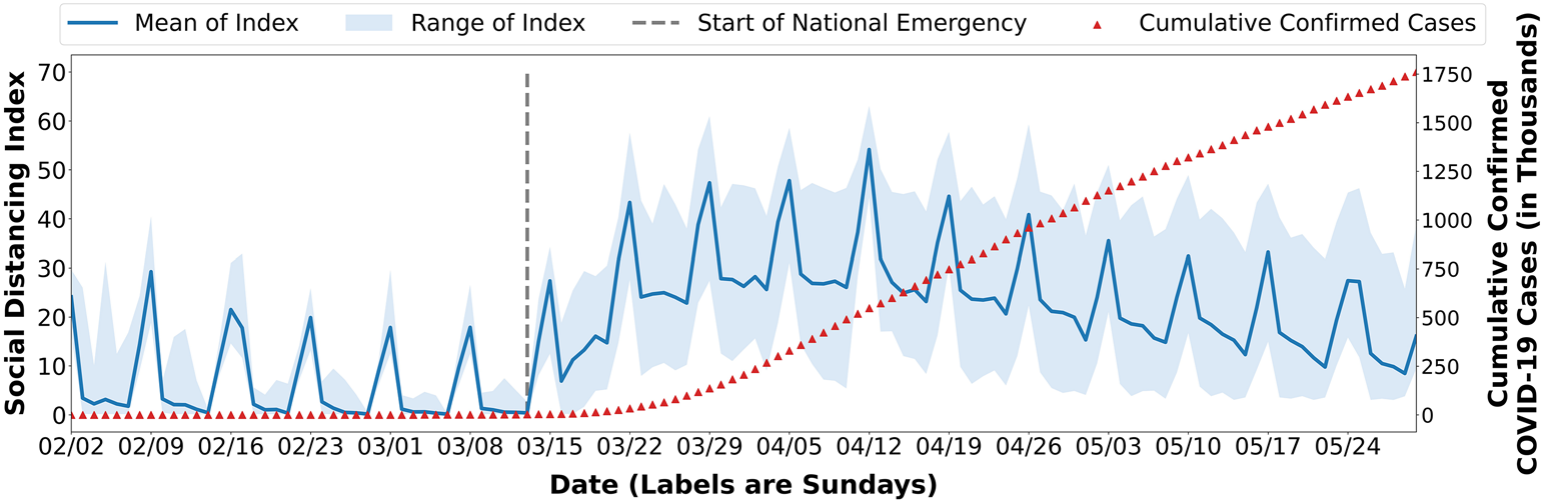
Temporal changes of state-level Social Distancing Index. Figure aggregates the temporal change of SDI for the fifty states and the District of Columbia. The blue line shows the mean value of the state-level SDIs and the blue shadow shows the overall range. The grey dashed line marks the national emergency declaration in the U.S. The red triangular dots stand for the daily cumulative number of confirmed COVID-19 cases.

We examine the effectiveness and reasonableness of the proposed SDI by reviewing its temporal change from February 2, 2020 to May 30, 2020 and the spatial variation by state for the entire nation (Fig. 1). The proposed SDI is sensitive to people’s behaviour changes and is capable of reflecting the mobility changes accordingly. The SDI changes clearly indicate that people stay home more and travel less on weekends, especially on Sundays, and people travelled less on Memorial Day (May 25) compared with a normal Monday. During the study period, people practiced significantly more social distancing nationwide after President Trump declared a national emergency concerning the COVID-19 outbreak. The national emergency declaration immediately triggered people’s responses on weekdays beginning March 16 and on weekends of the following weeks: March 22, March 29, and April 5. In addition, the range of index became wider after March 16, indicating that people from different states were having distinct responses to the national emergency announcement.

After the week of March 23, we observe a general plateau in terms of social distancing practice. Beginning April 6, there was a tendency towards less social distancing in some states. One week later, a similar trend appeared across the entire nation. The possible reasons are twofold. First, people became less attentive to the outbreak as the outbreak persisted. Moreover, because of the widespread economic impacts of the pandemic, some people can no longer afford to maintain social distancing. As people reduce social distance measures, there is no significant slowdown in the number of reported COVID-19 cases.

### State-level mobility pattern changes

**Figure 2:**
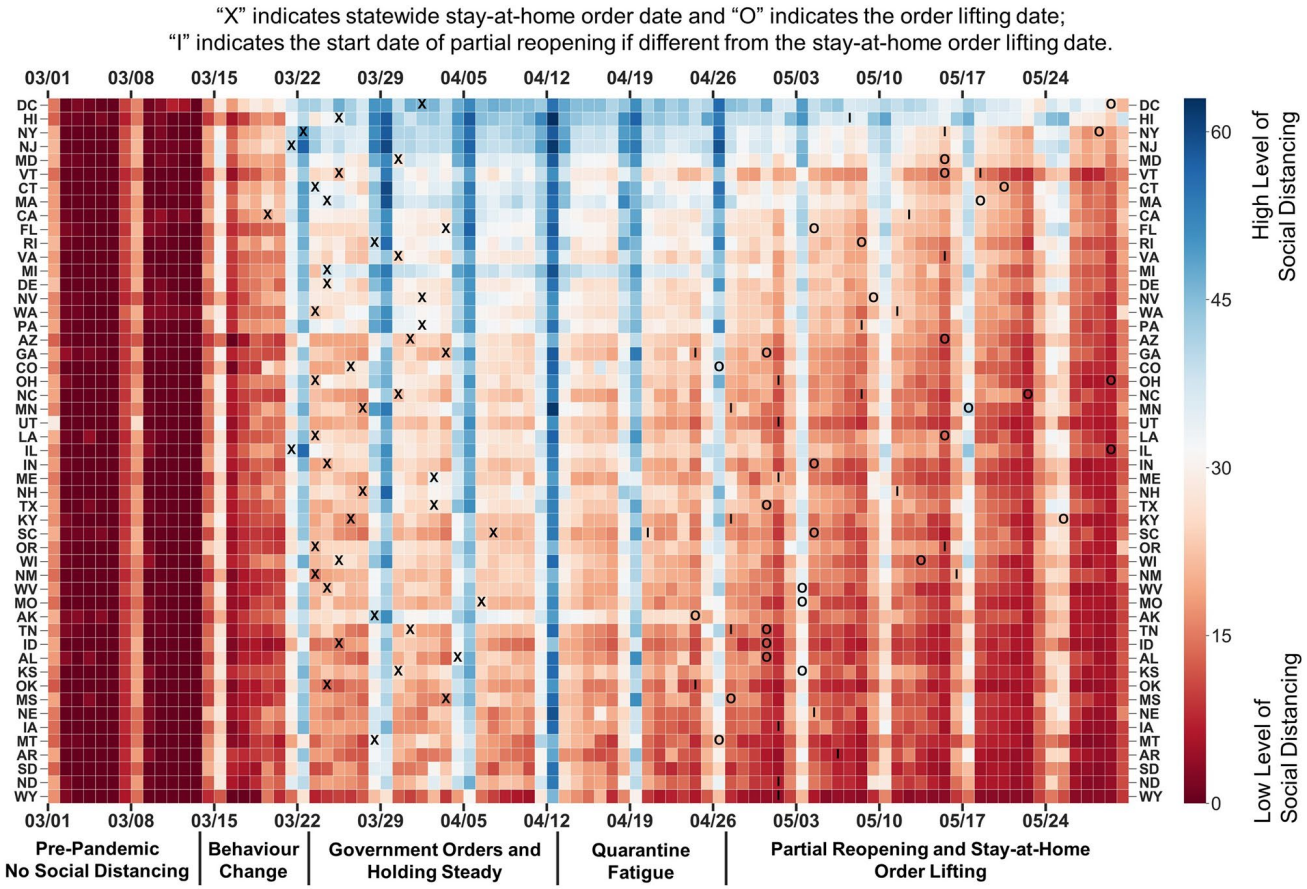
Social Distancing Index heatmap for all states. Figure shows the level of SDI scores for all states during the study period. Each pixel in the graph indicates the level of social distancing for one specific state on a specific day, where blue stands for more social distancing practiced and red for less. The “X” marker indicates the start date of state-wide, stay-at-home orders. The “O” marker indicates the order lifting date. The “I” marker indicates the start date of state-wide partial reopenings if different from the order lifting date.

Following the national emergency declaration, the mandatory stay-at-home orders issued by most states triggered a second wave of strengthened social distancing. This influence of government mandates on human behaviour can also be seen when some states began reopening: states that chose to lift stay-at-home mandates early saw an acceleration in social distance relaxation. The SDI is computed for all states for thirteen consecutive weeks from March 1 to May 30, 2020 in Fig. 2. Five stages are defined based on the general trend from all states: pre-pandemic (before March 13), behaviour change (March 14 to March 22), government orders and holding steady (March 23 to April 12), quarantine fatigue (April 13 to April 26), and partial reopening and stay-at-home order lifting (April 27 till now). The states are sorted in descending order by their SDI scores on the last weekday (May 29). The top five regions that are performing more social distancing are the District of Columbia, Hawaii, New York, New Jersey, and Maryland, all of which issued stay-at-home orders. Meanwhile, the states practicing less social distancing are Wyoming, North Dakota, South Dakota, Arkansas, and Montana, most of which did not issue stay-at-home mandates. On the East and West Coasts, it is possible that people practiced more social distancing because they were exposed to the infection risk for a longer period and are aware of the higher infection risk with higher population density.

**Figure 3:**
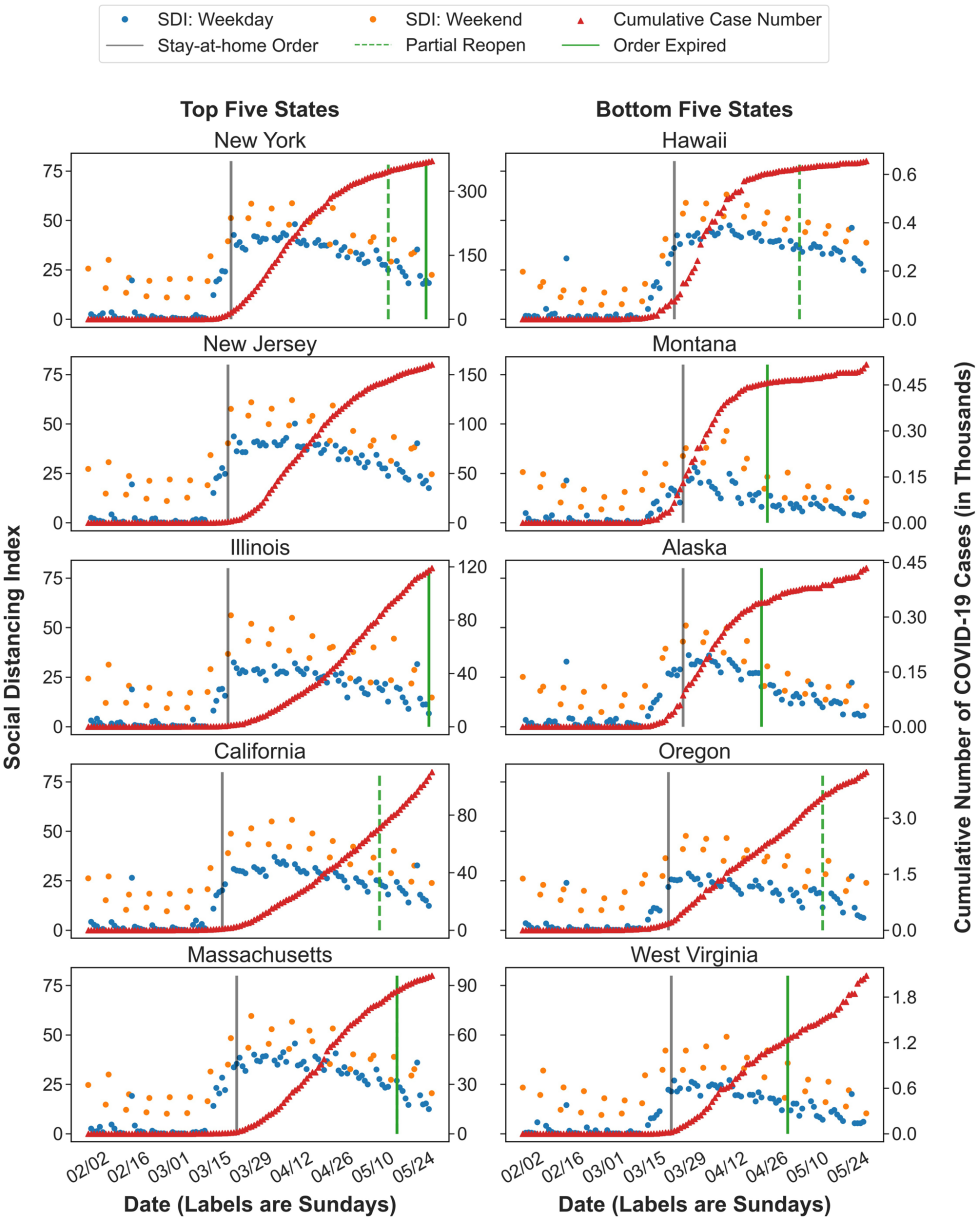
Temporal changes of Social Distancing Index in the top five and bottom five states regarding the cumulative number of confirmed cases. Figure demonstrates the temporal changes of SDI scores in the top five and bottom five states in terms of the cumulative number of confirmed cases on May 30, 2020. The blue dots stand for SDI scores on weekdays and the orange dots for SDI scores on weekends. The red triangular dots stand for the daily cumulative number of confirmed COVID-19 cases. The grey line stands for the start date of the state stay-at-home order. The green line marks the stay-at-home order lifting date and the green dashed line marks the date of state partial reopening.

In Fig. 3, we examine cumulative number of confirmed cases on May 30, 2020 for the top five and bottom five states. After the stay-at-home orders were issued, all 10 states experienced an increase in SDI, but the bottom five states generally had lower scores of SDIs. It implies that the local severity of the COVID-19 outbreak plays a significant role in people’s decision making. Although all ten states experienced a decrease in SDI after April 13, we observed a sharp decline following the partial re-opening and/or stay-at-home order lifting in New York, Massachusetts, and Alaska. It implies that people in those states were willing to maintain more social distancing for a longer period, but the early reopening discouraged social distancing behaviours. The influence of early reopening in Alaska appeared after two weeks when the increase in confirmed cases accelerated. Similar impacts of reopening can be observed in California, Montana, Oregon, and West Virginia, where the low level of SDI and increasing trend of confirmed cases raise concerns about a second local outbreak.

We also evaluated the Spearman’s rank correlation coefficients between the infection rates and the SDI scores for those ten states during the entire study period (Table 1). Since the SDI scores on weekends are systematically higher than those on weekdays, we only used weekday observations to compute the correlation coefficients. The cumulative infection rate is defined as the cumulative number of confirmed COVID-19 cases per thousand population, and the new infection rate as the number of new confirmed cases daily per thousand population. In Table 1, we observed a stronger correlation between SDI and new infection rate than that between SDI and cumulative infection rate, which implies that people were paying close attention to the outbreak development and have been practicing less social distancing. The stronger correlations between SDI and new infection rates in Hawaii, New Jersey, Massachusetts, and New York imply that people in those states were more attentive during the pandemic compared to other states. Those states also have a flatter curve of cumulative number of confirmed cases at the end of the study period.

**Table 1:** Spearman’s rank correlation coefficients between SDI and infection rates for the top five and bottom five states regarding the cumulative number of confirmed cases.

Top five states	Correlation between SDI and infection rate		Bottom five states	Correlation between SDI and infection rate	
	Cumulative	New		Cumulative	New
New York	0.546	0.645	Hawaii	0.643	0.711
New Jersey	0.571	0.655	Montana	0.495	0.574
Illinois	0.524	0.604	Alaska	0.506	0.597
California	0.525	0.623	Oregon	0.532	0.600
Massachusetts	0.549	0.652	West Virginia	0.522	0.611

Table displays the Spearman’s rank correlation coefficients between SDI scores and new and cumulative infection rates for the top five and bottom five states with regards to the cumulative number of confirmed cases on May 30, 2020.

### County-level mobility pattern changes

**Figure 4:**
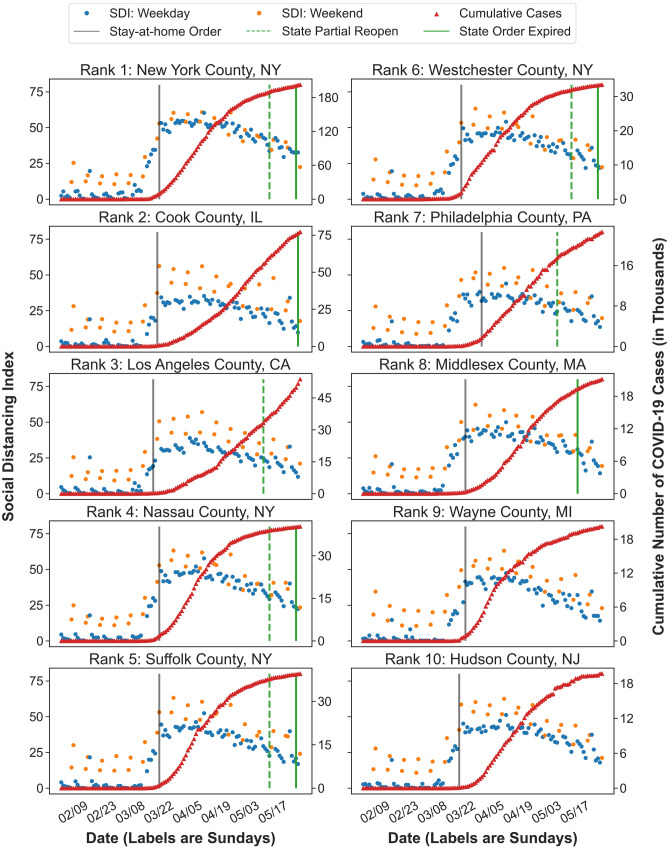
Temporal changes of Social Distancing Index in the top ten counties regarding the cumulative number of confirmed cases. Figure demonstrates the temporal changes of SDI scores in the top ten counties in terms of the cumulative number of confirmed cases on May 30, 2020. The blue dots stand for SDI scores on weekdays and the orange dots for SDI scores on weekends. The red triangular dots stand for the daily cumulative number of confirmed COVID-19 cases. The grey line marks the start date of state stay-at-home orders. The green line marks the stay-at-home order lifting date and the green dashed line marks the date of state partial reopening.

SDI is also informative at the county level. Figure 4 demonstrates the temporal changes of SDI for the top ten counties with regards to the cumulative number of confirmed cases on May 30, 2020. The counties in New York performed strict social distancing, which helped “flatten the curve” of cumulative confirmed cases. The high levels of SDI in Middlesex County, MA, Wayne County, MI, and Hudson County, NJ have also slowed down the outbreak. However, a relaxation of social distancing was observed after the partial reopening and the expiration of stay-at-home orders. The decreasing trend of SDI scores may change the trend in the near future. In the meantime, Los Angeles County, CA, and Philadelphia County, PA should strengthen social distancing as their SDI scores are lower than other counties in similar circumstances and their confirmed cases continue to increase at a rapid pace.

We evaluated the correlation between the infection rates and the SDI scores for the top ten counties with regards to the cumulative number of confirmed cases (Table 2). In general, we observed stronger correlations between the infection rates and the SDI scores in the counties with higher SDI scores. Moreover, the counties with smaller correlation coefficients between SDI and new infection rates tend to have an increasing trend in the cumulative number of confirmed cases at the end of the study period.

**Table 2:** Spearman’s rank correlation coefficient between SDI and infection rates for the top ten counties regarding the cumulative number of confirmed cases.

Top ten counties	Correlation between SDI and infection rate		Top ten counties	Correlation between SDI and infection rate	
	Cumulative	New		Cumulative	New
New York County, NY	0.589	0.696	Westchester County, NY	0.573	0.686
Cook County, IL	0.549	0.644	Philadelphia County, PA	0.579	0.647
Los Angeles County, CA	0.549	0.640	Middlesex County, MA	0.563	0.675
Nassau County, NY	0.571	0.672	Wayne County, MI	0.575	0.656
Suffolk County, NY	0.564	0.650	Hudson County, NJ	0.581	0.680

Table displays the Spearman’s rank correlation coefficients between SDI scores and new and cumulative infection rates for the top ten counties with regards to the cumulative number of confirmed cases on May 30, 2020.

## Discussion

During the COVID-19 pandemic, data-driven tools that can provide insight into human behaviour have been of paramount importance. In this paper, we introduced the real-world observations of human movements, i.e., mobile device location data, to study the impact of non-pharmaceutical interventions. By studying the travel behaviours of people across the United States, we developed a score-based Social Distancing Index (SDI) to capture people’s actual social distancing behaviours. Monitoring the SDI patterns, both spatially and temporally, enables policymakers to evaluate the effectiveness of related policies and to involve data-informed decision making for public health. In addition, SDI boosts public and community awareness regarding the ongoing situation for where they are living. People can use insights from SDI to evaluate the potential risks in their neighbourhoods.

Being exploratory research, this study could be further improved in several directions. Firstly, the basic mobility metrics could be generated considering regional differences. Specifically, the current definition of the stay-at-home population may introduce some bias due to different individual behaviours between residents in rural and urban areas. For example, many people living in rural regions still must make long trips to shop for essential goods while people in urban areas have a higher chance of obtaining essential items nearby (within 1.61 km from home) and thus are more likely to be identified as staying at home. Secondly, adding more mobility metrics to the SDI could contribute to the comprehensiveness of the index. For instance, the trip purposes could be inferred by integrating mobile device location data and point of interest (POI) data. Identifying where people visit could allow us to distinguish between essential and non-essential trips, in addition to distinguishing between work and non-work trips. Thirdly, variables measuring the relationship between human movements and disease transmission could be extremely valuable. Although it may be difficult to retrieve details such as contact tracing information from mobile device location data, the aggregate measurements can also be significant indicators, such as trips from and to heavily infected areas that yield potential exposure and disease transmission in the study area, on top of out-of-county trips that are currently included. Moreover, an expert survey on improving the weight assignments to different variables in SDI may also contribute to a better construction of the index if time allows. Observing the mobility patterns and COVID-19 evolution for a longer period may also shed light on the assignment of weights.

Another future research direction is to integrate SDI with existing epidemiological frameworks, such as compartmental models. A variable of interest in these frameworks is to understand how the input variables evolve during the course of the outbreak. Certain policies, such as mobility restrictions, can significantly reduce certain input variables like the reproduction factor of the disease. SDI can be employed in these models to enhance the input prediction in compartmental models.

## Methods

### Basic mobility metrics

For this study, the research team created a data panel by integrating multiple mobile device data sources representing person and vehicle movements to improve the quality of the data. The basic human mobility metrics are computed based on a set of peer-reviewed and validated algorithms^30^. The derived mobility metrics are also integrated with COVID-19 case data^31^ and population data^32^, and published in the University of Maryland COVID-19 Impact Analysis Platform^33^. The platform aggregates mobile device location data from more than 100 million devices across the nation on a monthly basis. Additional details can be found in another paper by the authors^30^.

Generated from the mobile device location data from February 2, 2020 to May 30, 2020, the five basic mobility metrics are defined and summarized in Table 3. The basic metrics are selected to cover the frequency, spatial range, and semantics of people’s daily travel.

**Table 3:** Definition and descriptive statistics (state-level) for the basic metrics.

Index	Metric	Description	Min	Max	Mean	Median
1	Percentage of residents staying home	Percentage of residents that make no trips more than 1.61 km away from home	13.0	58.0	26.1 SD: 7.6	25.0
2	Daily work trips per person	Average number of work trips made per person. A work trip is a trip going to or from one’s imputed work location	0.14	1.49	0.48 SD: 0.18	0.46
3	Daily non-work trips per person	Average number of non-work trips made per person	1.39	3.90	2.64 SD: 0.37	2.65
4	Distances travelled per person	Distances in kilometres travelled per person on all travel modes	15.6	113.4	52.3 SD: 14.3	52.1
5	Out-of-county trips (in thousands)	Number of all trips that travels from and to the outside of the county	7	28,845	5339 SD: 5299	3597

Table defines the basic human mobility metrics considered for the construction of SDI and summarizes the descriptive statistics of state-level estimates.

### Social Distancing Index

We designed the SDI as a score-based index, which gives a 0–100 score to each geographical area, e.g., a state or a county, and measures to what extent area residents and visitors practice social distancing. Zero indicates no social distancing and one hundred indicates perfect social distancing compared with the benchmark days before the COVID-19 outbreak. The benchmark values for the basic metrics are computed using data from the weekdays (Monday to Friday) during the first two weeks of February. Thereafter, the changes in people’s mobility patterns are captured by percentage reduction of the corresponding metrics in Table 1 (noted as ${X}_{2},\dots ,{X}_{5}$) as input. The absolute changes in the percentage of residents staying home (noted as ${X}_{1}$) also serve as input. The percentage reductions are absolute values between 0 and 100%. Any increase will be standardized as 0% in the calculation.

By jointly considering the travel behaviours of the region residents and visitors, the equation for computing SDI is given as follows:

1 $$SDI=\left[{\beta }_{1}{X}_{1}+0.01\times (100-{X}_{1})\times ({\beta }_{2}{X}_{2}+{\beta }_{3}{X}_{3}+{\beta }_{4}{X}_{4})\right]\times \left(1-{\beta }_{5}\right)+{\beta }_{5}{X}_{5}$$

where $${\beta }_{1}=1$$ and $${\beta }_{2}+{\beta }_{3}+{\beta }_{4}=1$$.

The first part of the equation focuses on resident travel and the second part on out-of-county trips. ${\beta }_{5}$ is thus the weight assigned to behavior changes regarding out-of-county trips. For resident trips, we use the percentage of residents staying home to account for residents that do not make trips longer than 1.61 km from home, so the weight is simply one (${\beta }_{1}=1$). For people not staying home (travellers), the percentage of which is $100-{X}_{1}$, we use a weighted sum of percentage reductions in the number of work and non-work trips made daily and the average distances travelled per person. When individuals make more work and non-work trips, and travel longer distances, they are considered to practice less social distancing. The weights for each variable should sum up to one (${\beta }_{2}+{\beta }_{3}+{\beta }_{4}=1$) so that the resident travellers are comparable to residents staying at home.

To assign appropriate weights to each variable, we have consulted both actual observations and conceptual guidelines. Firstly, we observe that the relative ratio between resident trips and out-of-county trips nationwide is about four to one. Hence, we assign a weight of 0.2 to ${\beta }_{5}$. Secondly, it is widely observed that people have significantly reduced travel distances so the index should not give the large percentage reduction in distances travelled the same weight as the reductions in number of trips. Meanwhile, the reductions in number of trips are more informative with regards to people’s reactions to the stay-at-home mandates. We thus consider the reduction in number of trips twice as important as that in distance travelled and assign a weight of 0.3 to ${\beta }_{4}$. Moreover, as suggested by government agencies, people are highly encouraged to reduce non-essential trips. The index should be designed to favor the reduction in non-essential trips, which is estimated twice as important as the reduction in essential trips. The work trips are intuitively considered as essential trips and non-work trips could include both essential and non-essential. Based on the 2017 National Household Travel Survey (NHTS) Travel Profile^34^, the relative ratio between essential and non-essential non-work trips is approximately 1:2. Therefore, the relative ratio between the percentage reduction of work and non-work trips is 1:1.67. According to the constraint ${\beta }_{2}+{\beta }_{3}+{\beta }_{4}=1$, we further assign 0.25 to ${\beta }_{2}$ and 0.45 to ${\beta }_{3}$. In this study, SDI is eventually computed as follows:

2 $$SDI=\left[{X}_{1}+0.01\times (100-{X}_{1})\times (0.25{X}_{2}+0.45{X}_{3}+0.3{X}_{4})\right]\times 0.8+0.2{X}_{5}$$

It should be noted that the weights are partially determined by certain assumptions. For example, the reduction of trips is more important than the reduction of travel distances when measuring the social distancing strength. We evaluated the sensitivity of SDI scores as the relative weights between the trip and distance reduction estimates changed. We found that the higher the weight assigned to the distance reduction estimates (${\beta }_{4}$), the larger the absolute values and standard deviations of SDI scores are, both spatially and temporally. When ${\beta }_{4}=1$, the largest absolute values and standard deviations of SDI scores are observed. Although the magnitude of SDI scores has changed, both spatial and temporal trends stayed the same in general. Therefore, such changes in weight assignments shall not yield to inconsistent inferences when comparing the social distancing practice between different regions and periods.

### Ethics declarations

The study was presented to and reviewed by the University of Maryland College Park (UMCP) Institutional Review Board (IRB). The study is exempt from the full board review as it only involves passive observation of public behaviour without collection of identifiable information, which falls into the federally-defined exempt categories (per Title 45 of the United States Code of Federal Regulations 46.104(d)). The authors only have access to de-identified data.

## Data Availability

The mobility metrics at the state and county levels, and the codes for computing Social Distancing Index (SDI) are published on Harvard Dataverse: Pan, Yixuan, 2020, "Replication Data for: Quantifying human mobility behavior changes during the COVID-19 outbreak in the United States", https://doi.org/10.7910/DVN/X25YT1, Harvard Dataverse.
